# Editorial: Insights in antimicrobials, resistance, and chemotherapy: 2021

**DOI:** 10.3389/fmicb.2022.1037326

**Published:** 2022-10-12

**Authors:** Rustam Aminov

**Affiliations:** The School of Medicine, Medical Sciences and Nutrition, University of Aberdeen, Aberdeen, United Kingdom

**Keywords:** antimicrobial resistance, drug target, antimicrobial peptides, phage therapy, antiseptics, animal models, digestive tract decontamination, combination therapy

Antimicrobial resistance (AMR) is a problem, which has emerged during the past several decades, and which is now familiar to many (Aminov, 2010). AMR infections require more expensive treatments and result in extended hospital stays and, most importantly, these infections claim human lives. Thus, there are continuous research and development efforts to counteract the current AMR crisis. The aim of this Research Topic was to gather some recent insights in this important area of research.

The vast majority of AMs in the current use target bacterial cell wall biosynthesis, bacterial membranes and replication, transcription, and translation machineries. Metabolomic approach opened new prospects in identification of novel bacterial targets for antimicrobials (AMs; Aminov, 2022). In this Research Topic, Khan et al. analyzed metabolic pathways of *Streptococcus pneumoniae* strains *via* the computational subtractive genomics approach to identify potential drug targets. In total, 47 potential drug targets were identified, with two of them, 4-oxalocrotonate tautomerase and sensor histidine kinase, being of particular interest, because they are unique to *S*. *pneumoniae*, and thus drugs targeting these proteins could be very specific.

Antimicrobial peptides (AMPs) are produced by many organisms from all three domains of life (Hao et al., 2022). In eukaryotes such as animals, AMPs comprise a part of their immune system in the form of innate immunity, while in bacteria and archaea as well as in lower eukaryotes they presumably protect their own ecological niches from invasion by other organisms. AMPs have attracted a considerable attention due to their AM potential. In particular, as noted by Martinenghi and Leisner, bacteriocins from lactic acid bacteria have been extensively studied during the past 35 years. While there has been, undoubtedly, a great progress in understanding the basic science of bacteriocins, applications of these findings, with the exception of food preservation, have been limited. The drawbacks of bacteriocins are in narrow target spectrum, target resistance, protease sensitivity, poor yields, and also in economic and regulatory hurdles. Potentials of AMPs in clinical or veterinary medicine have not been evaluated in large-scale clinical trials.

With the diminishing arsenal and efficiency of the current antimicrobials, however, AMPs have a potential to assist to at least some of the deficiencies of antimicrobial therapy. For example, AMs are less efficient against the biofilms that are formed by the majority of microbial infections. Bose et al. developed machine learning models for identification of AMPs with antibiofilm actives from a variety of sources. This *in silico* approach may help to identify new antibiofilm AMP leads and predict their antibiofilm efficacy.

It is a well-known fact that the vast majority of globally produced antimicrobials are used in agriculture (Van Boeckel et al., 2019). There is also a link, well-established within the One Health framework, between the antimicrobial use in agriculture and the rise of human AMR pathogens (Woolhouse et al., 2015). Thus, replacement of AMs in agriculture may have a considerable impact on reducing the rate of emergence and dissemination of AMR. Rodrigues et al. extensively reviewed potentials of AMs replacement by AMPs in agriculture. Presently, however, the use of AMPs in agriculture is limited due to low i*n vivo* efficacy, inadequate stability, and production costs. These deficiencies can be addressed *via* engineering of AMPs, association of AMPs with nanoparticles, and production optimization to reduce the cost.

Phage therapy has recently received a renewed attention as one of the possible antimicrobial alternatives (Abedon et al., 2017). Especially promising are the lytic components of bacteriophages that are potentially easier for accommodation under the current pharmacological regulations compared to native phage particles. One of these phage components are depolymerizes that degrade capsular polysaccharides, lipopolysaccharides and exopolysaccharides of the host bacteria thus making them more susceptible to antimicrobials and the immune system. Chen et al. investigated Dpo71 depolymerize from a lytic bacteriophage vB_AbaM-IME-AB2 that infects *Acinetobacter baumannii*. Dpo71 sensitized the multidrug-resistant (MDR) *A. baumannii* strains to the host immunity and increased their susceptibility to antimicrobials such as colistin. The downside of Dpo71, however, was in its narrow range, with the lack of activity against the phage-resistant *A. baumannii* strains. This limitation can be addressed using of cocktails of phage depolymerases or by engineering Dpo71 to broaden its host range and enhance its activity.

Similarly to any other drug, preclinical investigation of antimicrobial candidates in animal models is one of the prerequisites during the antimicrobial drug development. Animal models most frequently used during this stage are rodents, in particular mice. Two articles in this Research Topic discussed the role of murine models of lung infection in evaluation of AM efficiency. Review by Arrazuria et al. revealed pronounced variations in the experimental design of the murine pneumonia models. Importantly, differences in the immune status of animals, their age, infection routes, and sample processing had strong impact on the effect of AMs. Thus, preclinical models of disease need to be standardized to generate consistent and comparable results during the evaluation of AM candidates. Arrazuria et al. also came up with recommendations for a standardized design of preclinical murine pneumonia models that could help to harmonize the results obtained during the evaluation of novel AMs in different laboratories.

Antiseptics such as chlorhexidine digluconate (CHX) and cetylpyridinium chloride (CPC) are widely used in oral care products. Mao et al. revealed rather unexpected effects of these antiseptics, with CHX selecting for caries-associated saccharolytic taxa and CPC—for gingivitis-associated proteolytic taxa in oral biofilms. Antiseptic-resistant isolates were also resistant to various antibiotics. Thus, antiseptics in dental care products may have undesirable effects on oral microbiota, which warrant further investigations.

Selective Decontamination of the Digestive tract (SDD) is aimed at prevention of nosocomial infections by eliminating potentially pathogenic microbiota from the gastrointestinal tract (GIT). Buitinck et al. evaluated the SDD protocol with tobramycin, polymyxin B, and amphotericin B in patients colonized by at least one facultative aerobic Gram-negative bacterium on admission. SDD successfully eradicated the vast majority of susceptible and resistant bacteria from the upper and lower GIT. Eradication of AMR bacteria from the lower GIT, however, required a longer time period compared to patients colonized by susceptible bacteria.

Combination AM therapy is used to treat MDR infections. The fungal infections caused by *Candida* species become increasingly resistant toward the first-line anti-fungal drugs such as azoles that obstruct ergosterol biosynthesis by inhibiting the enzyme 14-alpha-demethylase. Liu et al. explored another target in *Candida*, heat shock proteins 90 (Hsp90). For this, they used a synthetic variant of geldanamycin, 17-Allylamino-17-demethoxygeldamycin (17-AAG), which belong to the benzoquinone ansamycins family of drugs, inhibiting ATPase activity of Hsp90. Although 17-AAG alone exerted a limited antifungal activity, its combination with azoles displayed synergistic effects.

Although the majority of AMR mechanisms are acquired horizontally, there is still a sizeable proportion of AMR due to chromosomal mutations. Li et al. investigated the daptomycin resistance mechanism in a clinical isolate of *Enterococcus faecium*. They established that the resistance is conferred by mutations in the chromosomal *cis* gene, which encodes cardiolipin synthase. These mutations led to the redistribution of lipids and to the decrease of surface negative charges in the cell membrane. In another study with daptomycin, Sulaiman et al. generated tolerance and resistance toward it in methicillin-resistant *Staphylococcus aureus in vitro*. Proteomic analyses of mutants suggested that daptomycin resistance was due to peptidoglycan changes, with a more positive surface charge to repel the antibiotic. But the tolerant phenotype displayed different cell wall changes, not involving peptidoglycan or surface charge alterations. Quinolone resistance emergence in *Escherichia coli in vitro* was evaluated by Perault et al. Repeated selection by a high ciprofloxacin concentration led to the emergence of *gyrB* mutants with a hyperpersistent phenotype but not a significant MIC increase. Interestingly, mutations were located outside of the canonical GyrB QRDR. Attention should be paid to the tolerant/persister variants since they are frequently the cause of therapeutic failure.

## Author contributions

The author confirms being the sole contributor of this work and has approved it for publication.

## Conflict of interest

The author declares that the research was conducted in the absence of any commercial or financial relationships that could be construed as a potential conflict of interest.

## Publisher's note

All claims expressed in this article are solely those of the authors and do not necessarily represent those of their affiliated organizations, or those of the publisher, the editors and the reviewers. Any product that may be evaluated in this article, or claim that may be made by its manufacturer, is not guaranteed or endorsed by the publisher.
